# Solutions-oriented research for sustainability: Turning knowledge into action: *This article belongs to* Ambio*’s 50th Anniversary Collection. Theme: Solutions-oriented research*

**DOI:** 10.1007/s13280-020-01492-9

**Published:** 2021-03-14

**Authors:** Maria Tengö, Erik Andersson

**Affiliations:** 1 Stockholm Resilience Centre, Stockholm University, 106 91, Stockholm, Sweden; 2 Unit for Environmental Sciences and Management, North-West University, Potchefstroom, South Africa

**Keywords:** Co-production of knowledge, Policy entrepreneurship, Solutions-oriented research, Sustainability science, Transdisciplinarity, Usable knowledge

## Abstract

In this perspective, we reflect upon the question: *what processes may help transition scientific insights on sustainability issues into practice and thus contribute to tackling the complex, systemic sustainability problems of today?* We use five forerunners in the field of providing and brokering knowledge for science informed real world solutions, all published in *Ambio* and highlighted in this Anniversary collection, as our starting point. We discuss how the authors present solutions, whom they tried to reach, and what was suggested—implicitly or explicitly—as the potential uptake processes for turning scientific knowledge into practice. With this as the starting point, we discuss how sustainability science, as a field vowed to action, has evolved in its views of actors, pathways for impacts, and the potential roles of research and researchers to promote sustainability transformations.

## Introduction

While scientific knowledge is vital for identifying essential components of potential solutions to environmental problems, it is often not sufficient in itself to turn ‘theoretical’ solutions real. In the light of the still ongoing and widespread misuse of the environment, and as abundantly clear from the increasingly numerous multi- and transdisciplinary studies of environmental issues, the belief that things will change as research and academic knowledge progresses is too simplistic, especially when looking at the major transformations needed to solve systemic and complex environmental problems (Díaz et al. 2019), such as those addressed in this anniversary collection: land use change and habitat restoration (Nepstad et al. 1991), aquaculture (Folke and Kautsky 1989), water consumption (Falkenmark 1989) and quality (Brix and Schierup 1989), and the use of nitrogen in agriculture (Cassman et al. 2002).

How, then, do you take academic knowledge to action and use it to support or spark change in a positive direction—and engage with the normativity of that last statement? How do you move from useful knowledge to knowledge-in-use in active learning, experimentation, and decision-making? Speaking to these points, there is today a growing literature on transdisciplinary knowledge processes. In these studies, solutions are sought jointly by researchers and practitioners (or other stakeholders), either throughout the whole research process or as an addition step once the research is ‘done’. This literature positions knowledge actors (researchers as well as others) brokering scientific knowledge as playing a vital role, and research is making it increasingly clear just what the role of brokering scientific knowledge may entail.

Focusing on this particular angle, we reflect on *how* the selected Anniversary Collection papers (Brix and Schierup 1989; Falkenmark 1989; Folke and Kautsky 1989; Nepstad et al. 1991; Cassman et al. 2002) describe the implementation and potential uptake processes for solutions to key sustainability issues (rather than the actual solutions proposed). More specifically, we look at how the five papers viewed the “receivers” of the solutions, and the suggested (explicit or more implicit) strategies for promoting uptake of scientific knowledge. Based on this we discuss and reflect on how the academia-society interface has evolved since the papers appeared in *Ambio* as front runners in delivering solutions-oriented sustainability science. We do this in pursuit of a tentative answer to the question *what processes may help transition scientific insights on sustainability issues into practice and thus contribute to tackling the complex, systemic sustainability problems of today?* Our outlook is grounded in a social-ecological systems perspective and draws on recent literature as well as our own experiences of transdisciplinary research in science-policy-practice interfaces.

## Some initial reflections on solutions as presented in the five papers

The five anniversary papers all target complex sustainability issues that remain challenging today, and they all point to the systemic nature of the problems: dryland water governance (Falkenmark 1989), socioeconomic factors behind tropical forest loss and regeneration (Nepstad et al. 1991), nitrogen management in agro-ecosystems (Cassman et al. 2002), aquaculture and more ecosystem-based food production (Folke and Kautsky 1989), and nature-based innovations and technology for improving water quality (Brix and Schierup 1989). These articles were novel in how they positioned sustainability problems and their potential solutions as embedded in and emerging from complex human-environmental interactions. In doing so, the five papers were front runners in the broad and diverse field of studies on natural resources management, ecosystem services, and nature’s benefits to people—and even more broadly, sustainability science.

In the rapid progress and expansion of the field of sustainability science, the five articles remain important in that they frame benefits as emerging from interactions between the social and the ecological—e.g., by viewing ecosystems as social-ecological systems, including social-technological infrastructures, cultural framings etc. The environmental problems analyzed in the papers are linked to and sometimes caused by clear societal needs—such as food production which is directly addressed in four of the five papers. This means that there will be trade-off decisions and different interests that need to be considered when devising strategies for engaging with the environmental problems.

Thus, to address these problems in practice, the scientific knowledge and understanding needs to reach and be incorporated into decision-making processes. The anniversary papers point to parallel policy processes and multiple lines of communication in their respective fields, including high-level reports, proceedings, extension work, and integrated research development projects. However, since none of the papers explicitly focused on knowledge uptake and implementation of the suggested solutions, we will use them here only as the starting point for discussing actors and mechanisms for impact, who the recommendation was meant for, and what were the envisioned pathways for implementing solutions?

## Who will implement the solution?

A critical aspect of delivering actionable knowledge is to identify who are its potential users and what decision-making context it needs to resonate with. The five papers differ in terms of how explicit they are in identifying and directly targeting specific actors or bodies in society as the intended agents for implementing the knowledge/solutions they present. Falkenmark (1989) is perhaps the one most clearly identifying a specific set of key actors for taking her recommendations to action: high-level national and international decision makers—African leaders, but also donor countries and the international organizations involved in development issues, in particular the United Nations. She portrays water issues, especially hydro-ecology, as a fundamental societal issue.

Folke and Kautsky (1989) outlined a strategy of combined cultivations of mussels and salmonids as a means to bring ecology back into what they saw as a highly artificial and environmentally costly aquaculture industry—i.e., they proposed a business solution rather than a change in policy. Brix and Schierup (1989) spoke mainly to implementation at the local level. They recommended ecosystem-based techniques for small villages, single farms, and small industries, but acknowledged the need for national and international decision makers to support the implementation. Cassman et al. (2002) targeted an information and knowledge gap concerning nitrogen use efficiency, from the farmers’ perspective as well as the researchers’, and argued that there are important roles to play by the private and public sector. In other words, while farmers were identified as the primary end-users or receivers, additional actors were mentioned as important facilitators and enablers of strong research and extension programs that can build capacity for efficient nitrogen use for food production. Overall, none of the papers offers much detail about who are the key actors that need to be involved.

Since the papers were written, the perception of who could (and need to) be involved in realizing different solutions to ensure uptake and more comprehensive use of knowledge has broadened and become increasingly nuanced. For example, where the biodiversity conservation and ecosystem management literature traditionally targeted managers with a formal mandate (like decision makers within government), there is now greater recognition of the need to interact also with a more diverse group of ‘users’, “informal” actors, or practitioners on the ground (Olsson and Folke 2001). Furthermore, this thinking about end-users and key actors for implementing solutions has now connected with the discourse on participation as a rights issue, and as a vehicle for further knowledge processing. Involving diverse actors offer ways to tap into complementary knowledge and create novel knowledge products (Tengö et al. 2017; Miller and Wyborn 2020).

A recent illustration of involvement of more diverse actors is the recognition of indigenous peoples and local communities (IPLC) and their knowledge in the global assessment report on biodiversity and ecosystem services of the Intergovernmental Science-Policy Platform on Biodiversity and Ecosystem Services (IPBES 2019; McElwee et al. 2020). The report cites evidence that at least a quarter of the global land area is used, owned, traditionally managed or occupied by indigenous peoples, and that these areas are often rich in biodiversity and with limited human intervention. The key messages for decision makers in this report include the need for conservation to build partnerships and recognize the validity of indigenous and local knowledge and governance systems as well as claims to territorial rights. Another example of a broadened perspective of who are key actors to engage with in Sustainability Science comes from the urban context. Urban studies are increasingly highlighting and promoting integration of perspectives and knowledge held by a wide array of actors (Colding and Barthel 2013; Andersson et al. 2014, 2017; Frantzeskaki and Kabisch 2016).

## What uptake processes do they identify or suggest?

The current literature on how to effect sustainability transformations or related solutions pathways thus recognize more actors and also expand the understanding of the actors themselves and the roles they may play to generate and implement ways forward. Actors carry care, knowledge and agency, which will all influence how they become part of realizing a solution (Andersson et al. 2017). In recognition of these different roles and the differences between actors, more attention has been given to dialog and to the knowledge creation process, when translating science-based knowledge into knowledge-in-use. This is indicated already in the five original papers and then expanded on by the authors in their reflections (this issue). Here, we view translation in the sense of Tengö et al. (2017) to entail a process of enabling mutual understanding between different actors.

The five anniversary papers are part of the emergence of sustainability science as a science that has vowed itself to action (Lubchenco 1998; Kates et al. 2001). This can of course be done in many ways—publishing in academic journal is not the only and very likely not the most efficient way for researchers to act on sustainability concerns. However, increasingly, the impact of knowledge and pathways to action has become part of the science. *Ambio* has during the previous 50 years contributed to this development, through encouraging and providing a venue for solutions-oriented research (see Lang and Wiek 2021, this issue). One could argue that the anniversary papers in different ways act to synthesize and ‘translate’ knowledge, in terms of making it relevant and accessible to decision-making. They do this by combining knowledge and understanding, often from different disciplines or across scales, in new ways, and then using this shift of perspective to identify or demonstrate concrete solutions for addressing the sustainability challenges. Falkenmark (1989) develops an index and visualization tool to better monitor and deal with risk and embeds it in clearly articulated strategies to handle looming water crises. The research of Brix and Schierup (1989) contributes to the development of an European Design Guide and Operations Manual on the use of Emergent Hydrophyte Treatment Systems. Still, the uptake of such knowledge products is often impeded by a combination of social values, political contexts, technological innovation and diffusion, and the obduracy of infrastructure and economic and institutional structures. This makes effective social, political and technological uptake and implementation uncertain, as recognized by the authors of several of the five papers. They described critical barriers to uptake and implementation of knowledge: Falkenmark (1989), for example, pointed to administrative barriers and concluded her paper with strategies to overcome these, in addition to the technical solutions needed.

However, beyond this recognition of barriers to awareness and uptake, the anniversary papers do not offer much further in terms of how to overcome barriers and create impact. It should be noted that the limited reflection on uptake processes does not necessarily mean that the authors were not engaged with such work. It may rather be a reflection of the contemporary views of what is considered publishable in scientific journals and who the intended readers are.

Until recently, the challenge of uptake and application of scientific knowledge to address sustainability has been viewed as a linear process of “bridging a gap”. In most transdisciplinary sustainability science today, the uptake of knowledge is viewed as a much more complex, an iterative process with interdependencies—that in itself is a topic of research. This shift in understanding is well illustrated in a recent paper by Cairney and Oliver (2020). The authors reviewed a wide-ranging literature on ‘barriers’ to academic knowledge having impact, and offer practical advice on how to respond to such barriers. However, in the second part of the paper, they reformulate the challenges and claim that to have impact, researchers need to be *policy entrepreneurs:* “To be a ‘policy entrepreneur’ is to find out where the action is, learn the rules of the game, form alliances, frame your evidence in relation to the dominant language of policy debate, and respond to socioeconomic context and events which help create windows of opportunity” (ibid, p. 238). This kind of engagement in increasingly acknowledged as important, and it is also something that itself is studied as an integral part of sustainability research (Westberg and Polk 2016; Roux et al. 2017).

In our experience, some tentative answers to the role of researchers for generating solutions to sustainability challenges lies in a plural way of thinking—i.e., to be part of, contribute to, and navigate multiple processes in the science-society interface. Some of these processes may be about contributing specific knowledge to an existing policy process, others about convening transformative spaces (Pereira et al. 2020), or about maintaining an iterative channel for dialog with key stakeholders (Roux et al. 2006). It may involve bringing in new knowledge or synthesizing existing knowledge, as exemplified by the five anniversary papers. In these processes, the researcher can play different roles, e.g., as a knowledge broker mobilizing, translating, and probing knowledge, connecting scales and issues (Tengö et al. 2017). Active engagement in knowledge processes, convened by the researchers themselves or other knowledge actors brokering scientific knowledge, require different skills and experiences than what is required in much of non-participatory research and education (Clark et al. 2016; Haider et al. 2018).

## Discussion: Convening—and studying—the interfaces

Where is solution-oriented research headed? The role of scientific knowledge generation and how it is connected to processes of governing natural resources has changed over the last decades, informed not least by a growing interest in research-in-practice and research *on* practice. The linear view of delivering knowledge to decision-making—and, in doing so, bridging a science-policy gap—has shifted toward an understanding that science, policy, and practices are not distinct sets of processes, but they are interlinked and co-evolving. Engaging in and convening diverse arenas for deeper collaboration are now seen as critically important for ensuring that scientific knowledge can be woven into the decision-making processes and actually matter in practice.

Actively facilitating the uptake of scientific knowledge means it has to meet other knowledge systems and through exchange, negotiation, and mutual learning be adapted to different decision-making contexts. This co-production of knowledge has emerged as a critical field for connecting research with action (Wyborn et al. 2019; Norström et al. 2020). From being a new approach to doing research of high relevance to society, it is now becoming an increasingly adopted approach for supporting the kinds of sustainability transformations that tend to be implicit in solutions to systemic problems (Wyborn et al. 2019). Concomitantly, new perspectives on who are key actors and who need to be involved in a social-ecological problem solving, as discussed above. Who is an expert, a stakeholder or a rights holder in a particular setting? How to engage with actors in different roles? What is the role of and challenges with collaborations between researchers and other actors in society? How can research interests be aligned with interests and demands of actual governance processes—while also keep space for novel perspectives and solutions? Collaborations need to be inclusive enough to be valid and just—but small enough to create spaces for mutual understanding and respect to develop. By necessity, these questions need to be addressed along with careful and transparent considerations of positionality, research ethics, and diverse socio-political contexts (Turnhout et al. 2020). This is particularly critical in a time of political populism, anti-science movements, and active misinformation campaigns (see e.g., Garcia et al. 2019).

Deeper, richer, and more continuous dialogs between scholars and other actors, beyond traditional education and extension work, would strengthen the path toward turning actionable knowledge into knowledge-in-use. More and more venues are emerging, but it remains challenging to find sufficient incentives for *all* actors to invest time and energy in this joint pursuit of legitimate and evidence-based solutions. Transparent practices and active discussions of validity and implications of research finding, including the uncertainties involved will need to be a critical component.

Finally, as stated by Ruckelshaus et al. (2020), a re-configured contract between science and society will require strong institutional support for researchers committed to activities in the interface. Research funders and other societal actors are increasingly requesting project designs where research and implementation are integrated for extensive strategies for communication and, implicitly, impact. However, in reality, it remains challenging to secure funding for the long-term collaborations and systems understanding, including socio-political settings, that is required. Also, outside of institutions with a strong identity as promoters of Sustainability Science, transdisciplinarity engagement will continue to depend on strong individual commitments.
